# Sustainable intensification through rotations with grain legumes in Sub-Saharan Africa: A review

**DOI:** 10.1016/j.agee.2017.09.029

**Published:** 2018-07-01

**Authors:** A.C. Franke, G.J. van den Brand, B. Vanlauwe, K.E. Giller

**Affiliations:** a Soil, Crop and Climate Sciences, University of the Free State, PO Box 339, Bloemfontein 9300, South Africa; b Plant Production Systems, Wageningen University, PO Box 430, 6700 AK Wageningen, The Netherlands; c International Institute of Tropical Agriculture (IITA), c/o ICIPE, PO Box 30772, 00100 Nairobi, Kenya

**Keywords:** Cereals, Nitrogen fixation, Phosphorus, Biotic factors, Residual benefits, Smallholder farmers

## Abstract

We conducted a systematic review of literature on the residual effects of grain legumes in cereal-based systems of sub-Saharan Africa (SSA) to quantify the magnitude and variability of rotational effects, to explore the importance of environmental and management factors in determining variability and to evaluate the evidence of the different mechanisms that explain rotational effects. We retrieved 44 unique publications providing 199 observations comparing continuous cereal performance with that of a grain legume-cereal rotation. The overall mean yield increase of 0.49 t grain ha^−1^, equal to an increase of 41% of the continuous cereal yield, is highly significant, but the variability in residual effects is large. Effects were more pronounced in southern Africa, the highlands of East Africa and the Guinea savannah, and less in the humid forest/derived savannah of West Africa and the Sudano-Sahelian zone. Maize showed stronger yield responses after a legume than millet and sorghum. Agro-ecological zone and cereal type were however confounded. All grain legume types significantly improved cereal yields, with stronger residual effects observed after soybean and groundnut than after cowpea. Fertiliser N application to cereals reduces the residual effects of legumes, but the response at 60–120 kg N ha^−1^ still equalled 0.32 t ha^−1^ or 59% of the response when no N is applied. The sustained benefits with large N applications indicate the importance of non-N effects. While mechanisms for improved soil P availability after grain legumes have been studied in some detail, it remains uncertain how important these are in farmers’ fields. Grain legumes are unlikely to have a major influence on the availability of nutrients other than N and P, or on soil pH. Beneficial impacts of grain legumes on soil organic matter content can occur if legumes contribute to a greater overall cropping productivity, but studies generally report no such impacts. Evidence of impacts of grain legumes on weeds is limited to striga. Studies on the impacts on nematode pressure in cereals are inconclusive, probably because legumes act as a host for some of the key nematode genera that harm maize. The impact on the pressure of other pests and diseases in cereals is probably important, but evidence on this from SSA is lacking. Future research on N_2_-fixation by grain legumes and residual N benefits should focus on explaining the wide variability observed among sites. There is a clear need for more detailed mechanistic studies to assess the occurrence and relevance of non-N effects of grain legumes, particularly in relation to common pests and diseases in cereals.

## Highlights

• We retrieved 44 publications and 199 observations comparing continuous cereal with grain legume-cereal rotation in SSA.
• Cereal after legume yielded on average 0.49 t grain ha^−1^ or 41% more than continuous cereal.
• Sustained residual benefits of legumes with large N applications indicate the importance of non-N effects.
• Relevant non-N effects include improved P availability, changes in SOM, and in pest, disease and striga pressure.

## 1 Introduction

Diversification and intensification through inclusion of grain legumes in cereal, root or tuber based cropping systems represents a key technology in the drive towards the sustainable intensification of agriculture in sub-Saharan Africa (SSA) (Vanlauwe et al., 2014). Grain legumes fix atmospheric nitrogen gas (N_2_) that can contribute to the nitrogen (N) economy of fields, provide other rotational benefits to subsequent crops, produce *in situ* high-quality organic residues with a high N concentration and a low C to N ratio, and thereby contribute to integrated soil fertility management (ISFM) (Giller, 2001, Vanlauwe et al., 2010). Their protein-rich food and feed products have a good market demand in SSA where marketing channels are available (Chianu et al., 2011). The wide range of grain legume crops and varieties with different growth durations and other characteristics suggest that legumes have a potential niche in a wide range of farming systems in SSA. Legume production may be enhanced by replacing cereals or other non-legume crops, by intensifying crop production (instead of fallowing land or including legumes as an intercrop with cereals), or by expanding the area of farmland. Quantifying the rotational effect of grain legumes on subsequent crops is important for understanding the adoption potential of legume technologies as well as their impact on sustainability of production. Grain legumes often yield less and demand more labour than cereal crops due to labour-intensive manual harvesting, threshing, weeding and sowing practices (Franke et al., 2010, Ojiem et al., 2014). The rotational effects of legumes on cereal yields may nevertheless make legume-cereal rotations more attractive in terms of productivity and economic performance than continuous cereal cropping (Franke et al., 2014). However, the impact of legumes on subsequent cereals is highly variable, depending on soil fertility status, agro-ecological conditions, crop type and management, which in turn are affected by farmers’ diverse socio-economic conditions (Ojiem et al., 2006). Quantifying and understanding the variability in rotational benefits will help in the tailoring of legume technologies to environments in SSA where they work best.

The various rotational effects of grain legumes on subsequent crops can be divided into ‘N-effects’ and ‘non-N-effects’, also known as ‘other rotational effects’. Nitrogen effects refer to the improved N nutrition of a subsequent non-legume crop and the associated reduction in N-fertiliser demand as a result of the N_2_-fixing capabilities of legumes. The amount of N_2_ fixed depends on the genetic potential of the legume, the rhizobia and the symbiosis, and on the ability of legumes to establish their symbiosis which depends on the environment and management (Giller, 2001). In case where most of the fixed N_2_ is removed at crop harvest, the field N balance of a legume crop is close to zero or even negative. Nevertheless, in such a situation more N may be available for the subsequent crop than after a cereal. This can be due to an N-sparing effect (the absence of soil N depletion compared with a cereal grown without sufficient N input) or reduced N immobilisation of soil mineral N due to the lower C-to-N ratio of legume residues (Chen et al., 2014). N-effects from legumes depend on the amount of N fertiliser applied to the subsequent crop and in general are more pronounced in N-poor than in N-rich environments. Comparing the nitrogen budget of a non-legume crop following a legume with that of continuous non-legume crops where little N is applied allows estimation of the N-effect in terms of additional yield or N uptake (Giller, 2001). However, this approach tends to overestimate the N effects, and it often remains impossible to assign an increase in yield or N uptake of a non-legume crop after a legume to an increase in N availability or to other rotational effects, or their interaction. Isotope dilution methods may be used to directly estimate the N carry-over to subsequent crops, but these approaches are open to multiple interpretations (Chalk et al., 2014).

‘Non-N-effects’ of legumes refers to impacts mediated by biotic factors such as the occurrence of pests, weeds and diseases, and abiotic factors such as changes in the availability of water or nutrients other than N, changes in soil pH, or changes in soil organic matter and soil structure. While many studies in SSA assessed N dynamics in legumes by measuring N_2_-fixation rates, legume field N balances and N uptakes by subsequent crops, the non-N-effects effects are often neglected. Nevertheless, the non-N-effects may be of great importance. For instance, in environments with intense striga pressure (*Striga asiatica* or *S. hermonthica*), a non-host legume crop can drastically reduce the striga seedbank leading to lower striga densities and strong yield increases of cereals in rotation relative to continuous cereal cultivation (Franke et al., 2006, Rusinamhodzi et al., 2012). Also in temperate climates, the impact of legumes on biotic factors can be as important as the N effects where soil N is limiting (Kirkegaard et al., 2008). However, the question remains how widespread such strong impacts of legumes on biotic constraints of subsequent crops are. Apart from biotic stresses, legumes may influence the activities of other rhizosphere organisms that stimulate or suppress plant growth or available nutrients. The biotic impact of pests and diseases occurring belowground are hard to quantify, which explains why they receive little attention in field studies. An additional complexity to quantify some of the non-N-effects of an abiotic nature is the slow rate at which they change. Impacts of crop rotations on soil fertility parameters such as soil organic matter contents, soil structure and water holding capacity are typically only visible in longer-term experiments including several cropping cycles, which are scarce in SSA.

We are unaware of any recent studies that synthesise and structure the knowledge on the rotational benefits of grain legumes in the (sub-)tropics. Given that much less fertiliser is used in SSA than in other regions of the world, the contributions of N_2_-fixation are particularly important (Giller et al., 2013). Here we review the literature on the rotational effects of grain legumes, with a specific emphasis on SSA. Cropping systems in SSA outside the humid forest zone tend to be dominated by cereals, particularly maize, millet, sorghum and rice, combined with root and tuber crops where rainfall is adequate (Dixon et al., 2001). Specifically we: 1) Quantified the magnitude and variability of rotational effects of legumes on subsequent cereals; 2) Explored the importance of environmental and management factors in determining variability in rotational effects; and 3) Evaluated the evidence of the different mechanisms that explain rotational effects of legumes on subsequent crops.

## 2 Methods

### 2.1 Literature search

**Fig. 1:**
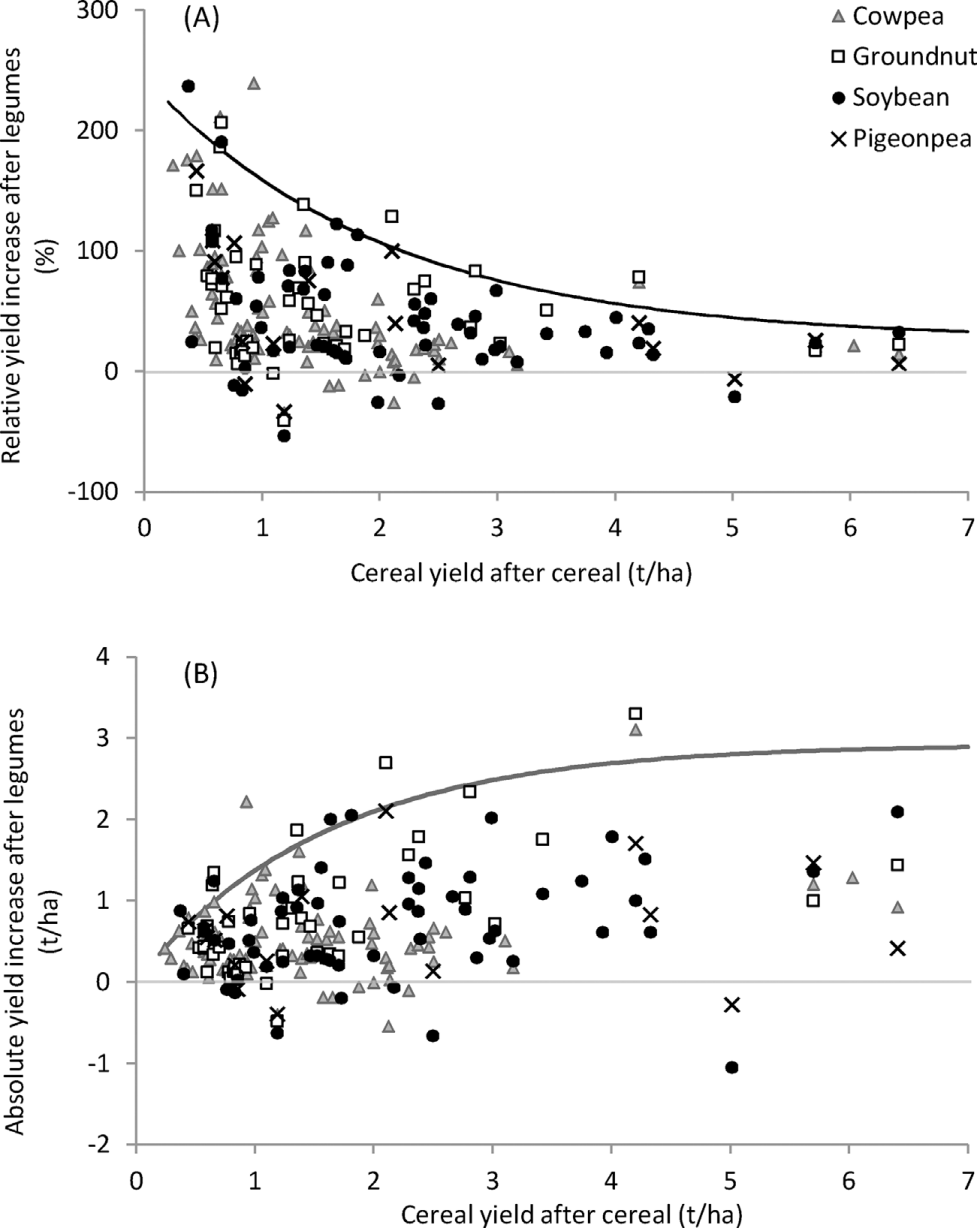
Relative (A) and absolute (B) grain yield increase of cereal after different types of legumes, relative to the yield of continuous cereal, plotted against continuous cereal grain yield. The curved lines indicate the upper boundary of maximum yield increases.

We systematically searched the Web of Science with the terms “legume* AND maize AND rotation”, “legume* AND sorghum AND rotation*”, “legume* AND millet AND rotation*”, “legume* AND rice AND rotation*” and “legume* AND rotation* AND Africa” and selected publications on experiments in SSA. Checking the references of the papers retrieved yielded six more papers. We also included a study from the current special issue. Only publications presenting primary source data from on-station or on-farm field experiments were included in the subsequent analysis. Legume-cereal mixed cropping experiments were excluded for two reasons: i) they are difficult to compare with monocrops of legumes and cereals and ii) the impacts on crop productivity depend strongly on spatial and temporal crop patterns. A requirement for inclusion of a study was that the cereal crops belonged to the same variety across treatments and were managed in the same way (including nutrient application rates). Where experiments were unbalanced, results from treatments were selected in such a way to ensure that treatments with cereal after cereal and cereal after legume were comparable. Where publications described experiments replicated across countries, districts representing different agro-ecologies, or different legumes or cereals, each comparison was considered separately. Similarly, if rotational cycles were repeated or N fertiliser treatments were applied to the cereal, results from individual cycles and N treatments were used. Results were averaged in case studies were conducted in multiple locations within the same districts or agro-ecology, or when studies included additional treatments such as different soil cultivation types or fertiliser applications to legumes. To compose the scatter graphs in Fig. 1 however, all individual treatments that could be extracted from the publications were used. To assess impacts per region, we divided studies from West Africa into three broad agro-ecological zones (AEZs): the humid forest/derived savannah (>1200 mm rain, >250 growing days per annum), the drier Guinea savannah (700–1200 mm rain, 150–250 growing days per annum), and the arid Sudano-Sahelian zone (<700 mm rain, <150 growing days per annum). Studies from Southern and East Africa were too few to be divided. A search on the Web of Science using the terms “tuber AND legume AND Africa” yielded few publications on legume-tuber mixed cropping, supporting the choice to focus our study exclusively on legume-cereal rotations.

To disentangle the different types of rotational effects of grain legumes on cereals, we extended our literature search with terms such as “N nutrition OR P nutrition”, “N fertiliser replacement”, “pest”, “weed”, “disease”, “soil structure”, “organic matter”, “pH”, “nematodes” in combination with “cereal* AND legume*”. Again, we focused on studies from SSA but in this case, studies from other parts of the world that provide insights into the mechanisms at play were also included. We reviewed estimates of the amount of nitrogen fixed and net N inputs by different grain legumes in SSA.

### 2.2 Data analysis

We tested for significant impacts of the factors: (1) agro-ecological zone (AEZ), (2) cereal type, (3) legume type, (4) N application rate to the cereal and (5) the number of rotational cycles on the mean cereal grain yield in continuous cereal, in legume-cereal rotations. As cereal crops, we distinguished maize, sorghum, millet and ‘other cereals’; legume crops were cowpea, groundnut, soybean, pigeonpea and ‘other legumes’. N application rates were categorised into no N applied, 15–50 kg N ha^−1^ and >60 kg N ha^−1^; the rotational cycles as 1 (1st rotational cycle) or >1 (2nd or higher rotational cycle). The precision of the reported means was expressed in the different publications as standard error of the mean (SEM), standard error of the (between-treatment) difference (SED), least significant difference (LSD), standard deviation (SD) and coefficient of variation (CV). In case SEM was not reported, it was calculated from available information on SED, LSD, SD and CV, as appropriate. In the subsequent analysis, the data were weighted inversely proportional to the square of the SEM in question (that is, inversely proportional to the variance of the mean). Studies that did not give an indication of variability (Sauerborn et al., 2000, Sanginga et al., 2002, Bado et al., 2006a, Bado et al., 2013, Bloem et al., 2009, Kamanga et al., 2010) were excluded from these statistical analysis. In the first instance, mean yields were analysed statistically using a mixed model with the fixed effects treatment (continuous cereal versus legume-cereal rotation) and cereal type, and the random effects pair, experiment x treatment and district x treatment. The factor “pair” matched each mean yield in legume-cereal rotation to the associated control yield (continuous cereal). In some experiments, mean yields for legume-cereal rotation were reported for different legumes, and/or for different levels of N and different rotational cycles. In order to model the potential correlation of treatment differences within experiments, the experiment x treatment random effect was fitted. Similarly, in order to model the potential correlation of treatment differences when experiments were carried out in the same geographic district, the district x treatment random effect was fitted. From the mixed model analysis, mean yields for the two treatments were calculated (continuous cereal versus legume-cereal rotation), as well as the difference between mean yields with associated 95% confidence interval and *p*-value. Similarly, mean yields for each cereal type and AEZ were calculated (SAS least squares (LS) means). Degrees of freedom and standard errors of estimates were adjusted using the Kenward-Roger (KR) method. In a sensitivity analysis, the mixed model analysis was repeated respectively fitting the treatment x cereal type and treatment x AEZ interaction terms. In the event, neither of these terms was statistically significant. Furthermore, in order to investigate the potential effect of legume type, a model was fitted with the fixed effects treatment, cereal type, legume type and treatment x legume type interaction. The potential effects of N application rate and of number of rotational cycles, were analysed in the same way. No inferential statistical analysis were made with regard to the impact of abiotic factors other than N application rate and of biotic factors, as the number of relevant papers on these topics did not allow such analysis

**Fig. 2:**
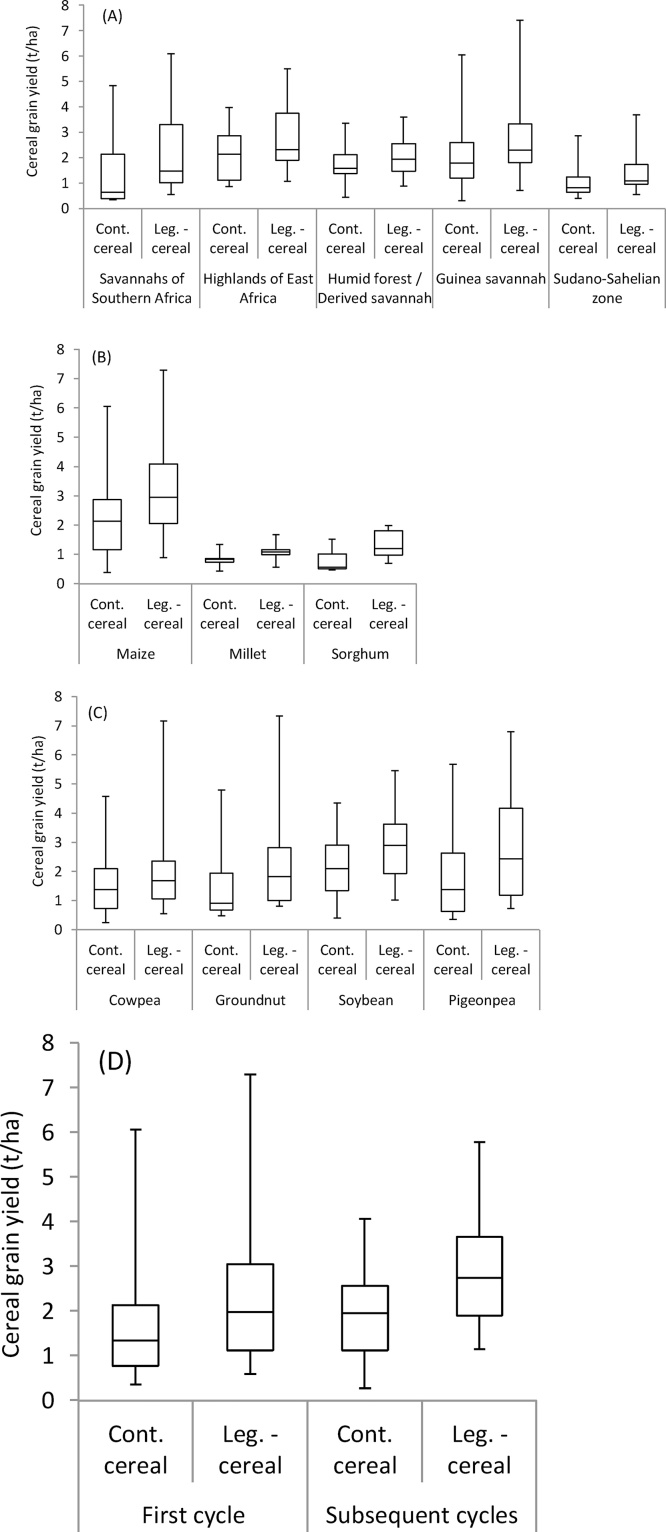
Box plots of cereal grain yield in continuous (cont.) cereal and legume (leg.)-cereal rotations separated by (A) the main agro-ecological zones, (B) cereal type, (C) legume type and (D) the number of rotational cycles. The whiskers represent the 2nd and 98th percentile of the observations.

**Fig. 3:**
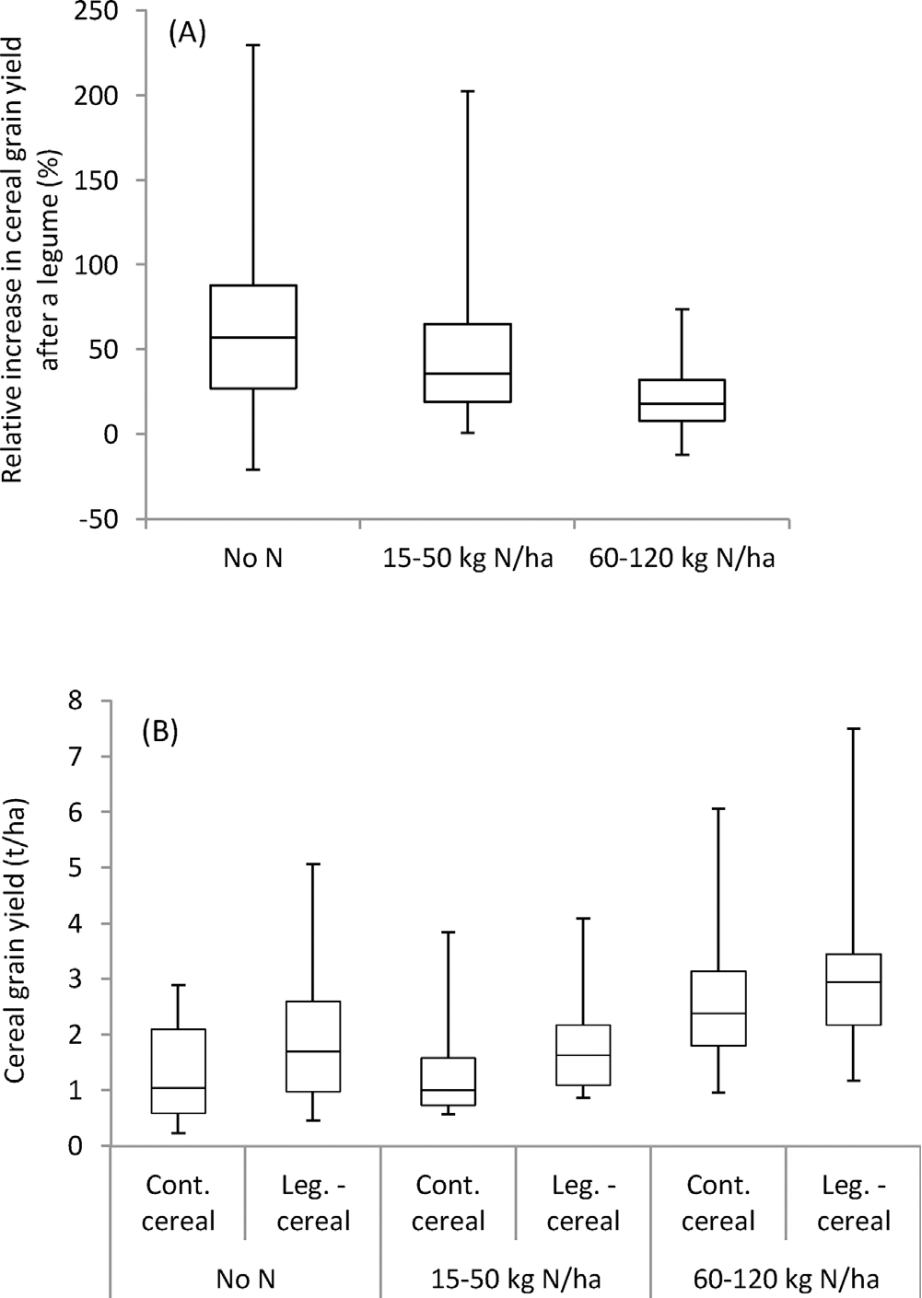
Box plots of (A) percentage grain yield increase of cereal after a legume crop, relative to the yield in continuous cereal, and (B) cereal grain yield in continuous (cont.) cereal and legume (leg.)-cereal rotations, at different N application rates to cereals. The whiskers represent the 2nd and 98th percentile of the observations.

Maximum response curves in Fig. 1 were obtained using boundary analysis (Shatar and McBratney, 2004) by regression of an exponential curve through the 95th percentile of yield responses after a legume (response variable) in the control yield ranges of [0–0.5], [0.5–1.0], [1.0–1.5], [1.5–2.0], [2.0–3.0], [3.0–5.0] and (>5.0 t ha^−1^), using the average control yields in these ranges as the explanatory variable. Means predicted by the statistical model and the associated *p*-values are presented in Table 3. Data presented in Fig. 1, Fig. 2, Fig. 3 and in Table 2 are based on the data as reported by the studies. ‘*n*’ in tables indicates the number of observations.

### 2.3 Overview of the available studies

We found 44 unique publications that assessed the impact of grain legumes on subsequent cereal yields in SSA (Table 1). These publications provided 199 comparisons between the cereal yield after a cereal and the cereal yield after a grain legume. Most studies were from West Africa (28 publications); within West Africa, most were conducted in the Guinea savannah (Table 1). Only one study was situated in the humid forest zone (southern Cameroon) (Jemo et al., 2006). No studies were found from Central Africa. In East Africa, studies were limited to the mid- to high altitude areas of Kenya and Uganda (1000–1600 m altitude, >1200 mm rain, two growing seasons and 250–365 growing days per annum). Studies from southern Africa were all conducted in the savannahs of the interior regions (600–1200 mm rain, a single growing season and 100–250 growing days per annum), except for one study (Bloem et al., 2009) conducted in the sub-humid east coast of South Africa (600–800 mm) which was excluded from analyses of effects of AEZs. Most studies (33 publications) focused on maize (*Zea mays* L.) as a rotational cereal crop, with fewer studies including sorghum (*Sorghum bicolor* [L.] Moench), millet (*Pennisetum glaucum* [L.] R. Br.), rice (*Oryza sativa* L.) or finger millet (*Eleusine coracana* L.) (Table 1). In the Sudano-Sahelian zone, only millet and sorghum were used as rotational crops.

**Table 1:** Number of unique publications (*n*) on rotational effects of legumes on cereals per AEZ and the countries where the trials were conducted and the cereal crops assessed. The number between brackets indicates the number of studies for a country/cereal crop. One publication contained data from both the derived and the Guinea savannah of West Africa, three studies included two cereals, one study contained observations from two countries.

Region	AEZ	*n*	Countries	Cereal crops
East Africa	Highlands	5	Kenya (4), Uganda (1)	maize (4), finger millet (1)
Southern Africa	Savannah and coastal region	11	Zimbabwe (5), Malawi (3), Mozambique (1), South Africa (1), Tanzania (1)	maize (10), sorghum (1)
West Africa	Humid forest/Derived Savannah	5	Ghana (2), Benin (1), Cameroon (1), Nigeria (1)	maize (5)
	Guinea Savannah	16	Nigeria (10), Ghana (4), Burkina Faso (2), Benin (1)	maize (13), sorghum (3), rice (1)
	Sudano-Sahelian zone	8	Burkina Faso (2), Mali (3), Niger (3)	millet (6), sorghum (3)

**Table 3:** Cereal grain yield in continuous cereal, cereal yield after a legume, yield response to a legume, and the significance of yield responses and of interactions between factors and yield responses, presented by AEZ, cereal type, legume type, the number of rotational cycles and N application category. Mean yields and yield responses (t ha^−1^), the associated standard errors (SE) and *p*-values were estimated by the statistical model.

Factor	*n*	Yield in continuous cereals	Yield after a legume	SE of yields	Yield response	SE of yield response	p-value
Overall mean	199	1.20	1.69	0.23	0.49	0.09	<0.0001
Savannahs of southern Africa	27	1.65	2.34	0.30	0.69	0.18	0.0005
Highlands of East Africa	30	2.24	2.86	0.32	0.62	0.21	0.0074
Humid forest/Derived savannah	22	1.59	1.81	0.25	0.21	0.21	0.334
Guinea savannah	74	2.14	2.66	0.21	0.52	0.18	0.015
Sudano-Sahelian zone	46	1.16	1.44	0.22	0.28	0.17	0.138
*AEZ x yield response*							0.351
Maize	131	1.96	2.53	0.15	0.57	0.12	0.0001
Millet	37	1.11	1.43	0.25	0.32	0.21	0.134
Sorghum	25	1.06	1.18	0.39	0.12	0.26	0.647
Other cereal	6	0.93	1.26	0.78	0.33	0.42	0.445
*Cereal type x yield response*							0.435
Cowpea	91	1.21	1.60	0.23	0.40	0.09	0.0004
Groundnut	35	1.22	1.84	0.23	0.62	0.11	<0.0001
Soybean	47	1.18	1.80	0.23	0.62	0.10	<0.0001
Pigeonpea	11	1.23	1.66	0.25	0.43	0.11	0.0005
Other legume	15	1.24	1.61	0.25	0.36	0.11	0.0023
*Legume type x yield response*							<0.0001
1st rotational cycle	150	1.49	1.97	0.13	0.48	0.10	<0.0001
2nd or subsequent cycles	49	2.08	2.57	0.16	0.49	0.12	0.0003
*Rotational cycle x yield response*							0.907
0 N	77	0.97	1.51	0.24	0.54	0.11	<0.0001
15–50 kg N ha^−1^	65	1.16	1.65	0.25	0.49	0.15	0.0025
60–120 kg N ha^−1^	53	1.92	2.25	0.26	0.32	0.14	0.0248
*N application x yield response*							0.204

## 3 Results and discussion

### 3.1 Magnitude and variability of cereal grain responses

#### 3.1.1 Overview of responses

An overview of the reported responses of cereal grain yields after a previous grain legume relative to continuous cereal cropping, averaged per legume crop, per district and over rotational cycles, is given in Table 2. In almost all cases, the grain yield of a cereal crop after a legume crop was greater than the grain yield of continuous cereal crops. The most common grain legume crops assessed were cowpea (*Vigna unguiculata* (L.) Walp.), soybean (*Glycine max* (L.) Merr), groundnut (*Arachis hypogaea* L.) and pigeonpea (*Cajanus cajan* (L.) Millsp.) (Table 2, Table 3). These were studied in all AEZs except for the Sudano-Sahelian zone where the focus was on cowpea and groundnut that are well adapted to drier conditions. Other legume crops included were common bean (*Phaseolus vulgaris* L.), Lima bean (*Phaseolus lunatus* L.), lablab (*Lablab purpureus* (L.) Sweet) and Bambara groundnut (*Vigna subterranea* (L.) Verdc.). Most studies assessed residual effects of several legume species simultaneously. The study by Rusinamhodzi et al. (2012) was excluded from further analyses of mean effects, as the extremely strong increase in maize yield after pigeonpea (≈5 t ha^−1^ or 800% increase), largely caused by a strong reduction in striga pressure, had a disproportionately strong influence on the results and was considered atypical.

**Table 2:** Overview of cereal grain yield response to a preceding legume relative to continuous cereal yield in the same season(s) (control yield). Results from additional treatments, sites within the same agro-ecology and different rotational cycles were averaged per publication. Eleven publications contained data on multiple cereal crops and/or districts and therefore occur more than once. The right columns indicates fertiliser treatments and to which crop nutrients were applied, and the number of rotational cycles. N fertiliser treatments are applied to the cereal unless mentioned otherwise.

AEZ/Source	Country	District	Cereal type	Control yield (t ha^−1^)	Change in cereal yield after legume (% relative to control yield)					Fertiliser treatment (crop)	No. of cycles
					Cowpea	Groundnut	Soybean	Pigeonpea	Other legume (type)		
*East Africa*											
Anyanzwa et al. (2010)	Kenya	Teso	maize	2.55			28			N	2
Kihara et al. (2010)	Kenya	Nyanza	maize	3.48			47			P (legume & maize)	2
Ojiem et al. (2014)	Kenya	Kakamega	maize	2.14		70	43		2 (common bean)/45 (Lima bean)/78 (lablab)	–	1
Ojiem et al. (2014)	Kenya	Siaya	maize	1.12		80	78		23 (common bean)/69 (Lima bean)/143 (lablab)	–	1
Ojiem et al. (2014)	Kenya	Vihiga	maize	2.87		68	41		5 (common bean)/50 (Lima bean)/103 (lablab)	–	1
Rao and Mathuva (2000)	Kenya	Machakos	maize	2.46	117					–	4
Ebanyat et al. (2010)	Uganda	Pallisa	finger millet	0.87	143	26	21	30	52 (green gram)	P (legume)	1

*Southern Africa*											
Kamanga et al. (2010)	Malawi	Dowa	maize	0.62					142 (pigeonpea/groundnut)	–	1
MacColl (1989)	Malawi	Lilongwe	maize	3.02		24	21			–	1
	Malawi	Dowa	maize	1.47			22			–	1
	Malawi	Mchinji	maize	2.30			56			–	1
	Malawi	Salima	maize	3.75			33			–	1
Rusinamhodzi et al. (2012)	Mozambique	Manica	maize	0.66				765		N & P (legume)	1
Bloem et al. (2009)	South Africa	Western Cape	maize	6.03	21					–	1
Bloem et al. (2009)	South Africa	Eastern Cape	maize	1.98	60					–	1
Marandu et al. (2013)	Tanzania	Muheza	maize	0.34	92			87	92 (green gram)	–	1
Kasasa et al. (1999)	Zimbabwe	Harare	maize	1.81			113			–	1
Kasasa et al. (1999)	Zimbabwe	Hurungwe	maize	0.37			237			–	1
Mupangwa et al. (2012)	Zimbabwe	Matobo	maize	1.11	119					–	1
Ncube et al. (2007)	Zimbabwe	Matobo	sorghum	0.52	131	131		123	82 (Bambara groundnut)	–	2
Thierfelder et al. (2012)	Zimbabwe	Shawva	maize	2.25	21					–	4
Zingore et al. (2008)	Zimbabwe	Murewa	maize	0.40			25			–	1

*West Africa − Humid forest/Derived savannah*											
Diels et al. (2006)	Benin	Sekou	maize	1.57	15			58		N	5
Jemo et al. (2006)	Cameroon	Southern Cameroon	maize	1.48	57		35			–	1
Adjei-Nsiah et al. (2007)	Ghana	Brong Ahafo	maize	1.76	15			58		N	1
Adjei-Nsiah et al. (2008)	Ghana	Brong Ahafo	maize	1.19	42					N	1
Franke et al. (2008)	Nigeria	Oyo State	maize	2.52			4			N	3

*West Africa − Guinea savannah*											
Oikeh et al. (2010)	Benin	Couffa and Dongo	rice	0.99			36			–	1
Bado et al. (2006a)	Burkina Faso	Houet	sorghum	0.64	211	186				N, P, K, Ca and Mg (legume)	1
Bado et al. (2013)	Burkina Faso	Houet	sorghum	1.52	29	22	18			N	1
Dakora and Keya (1997)	Ghana	Tolon-Kumbungu	maize	3.15	101	104		70		N	1
Dakora et al. (1987)	Ghana	Tolon-Kumbungu	maize	1.40	37	58				N	1
Horst and Härdter (1994)	Ghana	Tolon-Kumbungu	maize	2.36	29					P (maize)	2
Sauerborn et al. (2000)	Ghana	Tolon-Kumbungu	maize	0.65	151	207	191			–	1
Sauerborn et al. (2000)	Ghana	Tolon-Kumbungu	sorghum	0.57	65	77	117			–	1
Carsky et al. (1999)	Nigeria	Bauchi state	maize	1.66^a^	26					N	1
Carsky et al. (1999)	Nigeria	Kaduna state	maize	1.35^a^	10					N	1
Carsky et al. (2001)	Nigeria	Kaduna state	maize	0.27^a^	136					–	3
Franke et al. (2008)	Nigeria	Kaduna state	maize	2.03			91			N	3
Horst et al. (2001)	Nigeria	Kaduna state	maize	6.06	18	20	28	16	8 (common bean)	P (legume & maize)	1
Horst et al. (2001)	Nigeria	Plateau state	maize	3.15			−11	31	12 (common bean)	P (legume & maize)	1
Kolawole et al. (2007)	Nigeria	Kaduna State	maize	2.39			22			–	1
Oikeh et al. (1998)	Nigeria	Kaduna state	maize	4.20			24			–	1
Sanginga et al. (2002)	Nigeria	Niger state	maize	1.22			71			–	1
Yusuf et al. (2009b)	Nigeria	Kaduna state	maize	2.01	32		47			N	1

*West Africa − Sudano-Sahelian zone*											
Bado et al. (2006b)	Burkina Faso	Kouaré	sorghum	0.47	101					–	1
Bagayoko et al. (2000)	Burkina Faso	Gourma	sorghum	0.47	26					–	3
Bagayoko et al. (1996)	Mali	Ségou	millet	1.43	24					–	1
Kouyaté et al. (2000)	Mali	Ségou	sorghum	1.39	17					–	8
Kouyaté et al. (2000)	Mali	Ségou	millet	1.15	25					–	8
Samaké et al. (2006)	Mali	Mopti	millet	0.43	36					–	1
Bagayoko et al. (2000)	Niger	Dosso	millet	0.74	12					–	3
Bagayoko et al. (2000)	Niger	Tillabéri	millet	0.94	24					–	3
Bationo and Ntare (2000)	Niger	Tillabéri	millet	0.77	39	17			10	–	1
Bationo and Ntare (2000)	Niger	Tillabéri	millet	0.61	84	66			20	–	1
Bationo and Ntare (2000)	Niger	Dossa	millet	0.84	29	18			−7	–	1
Klaij and Ntare (1995)	Niger	Niamey	millet	0.43	29					–	3

a No continuous cereal included so the fallow-cereal treatment was used as the control.

#### 3.1.2 Impacts of control yield, AEZ, and cereal and legume type

The absolute response of cereals to rotation with grain legumes, as estimated by the statistical model, was 0.49 t ha^−1^, equal to a 41% increase relative to the continuous cereal yield (Table 3). The largest relative increases in grain yields in a cereal crop following a legume were observed when the control yield of continuous cereals was small, and declined exponentially with larger control yields (Fig. 1A), as also observed by Vanlauwe et al. (2001) for different maize-based systems in West Africa responding to legume and other organic inputs. Assuming the control yield in continuous cereal is an indicator of soil fertility, this suggests that under poor soil fertility the impact of growing a preceding legume can be large. Yet when constraints not addressed by rotation with legumes are overriding, the impact can be minimal. Absolute increases in cereal yield after a legume were overall largest with continuous cereal yields above 2 t ha^−1^ (Fig. 1B). At large control yields, absolute yield responses to the incorporation of a legume may still be substantial (Fig. 1B). Observations of absolute yield increases with control yields above 3 t ha^−1^ were too few to estimate reliably potential maximum yield responses when control yields were large.

Grain yields of cereals varied among AEZs (Fig. 2A & Table 3). Generally, cereals yielded poorly in the Sudano-Sahelian zone, presumably due to low and erratic rainfall leading to soil moisture stress. Smaller mean yield increases that were not significant were observed after grain legumes in the Sudano-Sahelian zone, as well as the humid forest/derived savannah of West Africa. Studies from the Guinea savannah of West Africa and from southern Africa and East Africa reported larger, significant cereal yield responses to rotation with legumes. The regional differences in responses to a preceding legume reflect the production potential of the relevant grain legumes in the regions. In the Sudano-Sahelian zone, the productivity of legumes is generally small due to the short growing season and frequent occurrences of drought, limiting the residual impacts on a subsequent cereal, and cereal yields were likely also to be constrained by water availability (e.g. Kouyaté et al. (2000)). Grain legume production is often poor in the humid forest/derived savannah due to moist and warm conditions leading to strong biotic pressures (e.g. Franke et al. (2008)). While rainfall is plentiful in the highlands of East Africa, the production environment is favourable for legume crops due to the relatively cool temperatures.

Differences in cereal yields among the AEZs were strongly influenced by the cereal crop grown (Fig. 2B & Table 3). In the Sudano-Sahelian zone of West Africa, only millet and sorghum were evaluated, while maize dominated in the other zones where 86% of the observations were on maize. The yield responses of millet and sorghum to a preceding legume were smaller and not significant, in contrast to maize showing larger and significant responses (Fig. 2B & Table 3). All millet trials were conducted in the Sudano-Sahelian zone that has a relatively low production potential for both legumes and cereals. Moreover, millet in the Sudano-Sahelian zone gives rather variable responses to N fertiliser (Traore et al., 2015), and the N benefits of growing legumes to millet may also be inconsistent. Studies on sorghum were conducted across different agro-ecological zones in SSA, with most observations from the Guinea savannah, and poor sorghum yields were observed across all areas, suggesting that sorghum has a smaller potential yield than maize. Due to the strong confounding between AEZ and cereal type, interactions between AEZ and yield responses and between cereal type and yield responses were not significant (Table 3).

All legume types had a significant residual effect on cereal yield (Fig. 2C; Table 3). A significant interaction between legume type and yield response was observed, and the mean effects from soybean and groundnut were larger than from cowpea. Nevertheless in some cases, strong residual effects of cowpea were reported (Horst and Härdter, 1994, Horst et al., 2001, Thierfelder et al., 2012). Cowpea has the ability to grow well in poor sandy soils and dry environments and the poorer response of cereals to a preceding legume may be related to the more marginal environments in which cowpea is often grown. While some general differences between legume crops in their ability to fix N_2_ and the net N contribution can be identified (see Section 3.2.1), N_2_-fixation is also strongly influenced by environmental and varietal characteristics, and differences in residual effects between legume types are not necessarily a reflection of N_2_-fixing abilities.

The number of rotational cycles did not affect the cereal yield response to a legume (Fig. 2D; Table 3). While the benefits of incorporating legumes for cereal yields are expected to increase over time, the number of studies reporting data over multiple rotational cycles was too small to observe this (Table 2). Cereal yields increased after the first rotational cycle in both continuous cereal and cereal after a legume. This might be the result of researchers and field technicians improving their skills in trial implementation over time.

#### 3.1.3 Interactions with N and P fertiliser

The application of 15–50 kg N ha^−1^ reduced the relative response of cereals from 56% (no N applied) to 42%, and applying 60–120 kg N ha^−1^ further reduced it to 17% (Fig. 3 & Table 3). Also the absolute response declined from 0.54 to 0.32 t ha^−1^ from no N to 60–120 kg N ha^−1^ applied. Nevertheless, the yield response to a preceding legume was significant in all N application classes (Table 3). If we assume that applying 60–120 kg N ha^−1^ strongly reduces or eliminates N as a limiting factor of cereal growth under smallholder conditions in SSA, the considerable response of cereals at 60–120 kg N ha^−1^ indicates the importance of other (non-N) rotational effects of legumes. When assuming that N-effects and non-N-effects are cumulative, the response at 60–120 kg N can be used to partition the response at 0N into non-N effects (equal to the response at 60–120 kg N or 59% of the total response at 0N) and N effects (the remaining response or 41% of the total response at 0N). It is likely however that N-effects and non-N-effects act synergistically. An improved availability of N to a cereal as a result of a preceding legume can lead to non-N-effects (e.g. an improved disease tolerance), while the non-N-effects of legumes may lead to better N recovery by a cereal.

Only few studies assessed interactions with P fertiliser in continuous cereal and legume-cereal systems in SSA (Horst and Härdter, 1994, Horst et al., 2001, Ebanyat et al., 2010, Kihara et al., 2010, Rusinamhodzi et al., 2012). Although some legumes have the ability to mobilise poorly-available soil P sources (see Section 3.2.2), additional P fertiliser is often essential for good grain legume growth and N_2_ fixation (Ogoke et al., 2003, Ronner et al., 2016). Better growth of a legume and the residual effects of P fertiliser applications can lead to enhanced residual effects in a subsequent cereal (Horst et al., 2001). P fertiliser may also be applied directly to the cereal crop, thereby enhancing growth and N use efficiency (Kihara et al., 2010).

### 3.2 Abiotic factors

#### 3.2.1 Nitrogen

Table 4 provides a summary of studies assessing N_2_-fixation and net N benefits from grain legumes in SSA. Most studies have been conducted on cowpea, groundnut and soybean. The range in grain yield of the common grain legumes is comparable with maximum yields up to 2.1–2.9 t grain ha^−1^. However, smaller maximum yields were reported for common bean, lablab and Bambara nut, perhaps due to the fewer studies conducted. All grain legumes have the potential to derive a large proportion of plant-derived N from atmospheric N_2_ and fix substantial amounts of N_2_ (often over 100 kg N ha^−1^), with the exception of common bean that is often, though not always, a relatively poor N_2_-fixer (Graham, 1981, Giller, 1990). Bambara nut had only modest biomass production in the few studies available and therefore fixed relatively low amounts of N_2_. The N harvest index of any grain legume is small when grain fill is hampered due to drought, heat stress or nutrient limitations. Soybean and common bean potentially have high N harvest indices (Table 4), because of the high protein concentration of the grains. However, as leaves of many soybean and common bean varieties senesce and fall before harvest, high N harvest indices reported in the literature may be partly due to an underestimation of the N returned to the soil before harvest. The net N input from N_2_-fixation, assuming stover remains in the field and grain is removed, and considering aboveground plant parts only, can be negative when the proportion of N derived from N_2_-fixation is small and/or most of the N is removed in the grain (Giller and Cadisch, 1995). The largest net N input rates are found when N_2_-fixation rates are high and the N harvest index is poor, for instance with indeterminate varieties. The large range in values of N_2_-fixation parameters within species reported in Table 4 is due largely to variability in environmental conditions, e.g. agro-ecological conditions (Ojiem et al., 2007), and management, e.g. P fertiliser applications (Ogoke et al., 2003), as well as the wide range of varieties available for most grain legumes. Varieties differ in duration, ability to nodulate with rhizobia present in the soil, productivity, dry matter and N harvest index, all of which affect the net N input from legumes. In general, longer duration, more indeterminate varieties with a high stover biomass production (also called dual-purpose varieties) fix more N_2_ and provide more residual N and soil fertility benefits (Mapfumo et al., 1999, Sanginga, 2003, Singh et al., 2003), though smallholder farmers often prefer shorter duration, more determinate grain-type varieties with a high dry matter and N harvest index (Snapp and Silim, 2002, Adjei-Nsiah et al., 2008). The additional N taken up by a cereal after a legume (Table 4) can be the result of an enhanced N supply and of an increased N demand due to other rotational effects. Only a limited fraction of the N in legume residues is available for uptake by the subsequent cereal. Laberge et al. (2011) using labelled ^15^N techniques at a site in the Sudan savannah reported that 40% of the N in cowpea residues remaining in the field could be retrieved in the top 30 cm of the soil at the beginning of the next planting season and 10% of the residual N was taken up by a subsequent millet. Sanginga et al. (2002) found that, at a site in the Guinea savannah, 17–33% of the N in soybean residues is taken up by a subsequent maize crop, depending on maize cultivar. Despite this low fraction of legume N in residues available for uptake by a subsequent cereal, the ranges in net N input from N_2_-fixation and additional N taken up by a subsequent cereal are comparable (Table 4), probably because legumes also enhance N demand of the subsequent cereal through other rotational effects. Some studies estimated an N fertiliser replacement value of legumes (Table 4). This value indicates how much N fertiliser should be added to a cereal following a cereal to produce the same grain yield achieved by a cereal in rotation with a legume without N fertiliser applied to the cereal. N replacement values are generally in line with the observed net inputs from N_2_-fixation.

**Table 4:** Observed ranges in yields, amounts of N_2_ fixed and net N inputs by different grain legumes in SSA grown as sole crops, considering aboveground plant parts only and assuming stover remains in the field and grain is removed (updated from Giller et al., 1997).

Grain legume	Grain yield (t ha^−1^)	Stover yield (t ha^−1^)	N from N_2_ fixation (%)	Amount of N_2_ fixed (kg N ha^−1^)	N in stover (kg N ha^−1^)	N harvest index (%)	Net input from N_2_-fixation (kg N ha^−1^)	Additional N taken up by subsequent cereal (kg N ha^−1^)	N fertiliser replacement value (kg N ha^−1^)
Cowpea a	0.1–2.7	0.3–2.1	18–96	4–201	19–150	21–49	6–125	2–59	10–80
Groundnut b	0.2–2.1	1.0–3.1	19–79	10–124	50–145	14–45	−11–43	7–73	60–67
Soybean c	0.3–2.4	1.1–3.9	9–96	3–302	4–110	28–84	−62–89	10–77	6–65
Pigeonpea d	0.1–2.9	0.3–7.9	28–100	1–97	9–103	5–44	0–82	10–33	19
Common bean e	0.3–1.3		3–56	1–31	8–19	34–87	−25–2		
Lablab f	0.1–1.4		48–72	54–172	41–205	9–22	0–131		
Bambara nut g	0.1–0.6	1.0–3.2	35–72	21–68	20–99	3–35	8–57	5–24	33

a Adjei-Nsiah et al. (2008); Dakora et al. (1987); Rusinamhodzi et al. (2006); Ojiem et al. (2007); Pule-Meulenberg and Dakora (2009); Belane and Dakora (2010); Nyemba and Dakora (2010); Bado et al. (2006a); Chikowo et al. (2004); Makoi et al. (2009); Vesterager et al. (2008); Ncube et al. (2007); Laberge et al. (2011); Naab et al. (2009); Jones (1974); Anyanzwa et al. (2010); Yusuf et al. (2009b); Adeboye et al. (2006); Kaleem (1989); Horst and Härdter (1994); Marandu et al. (2013).

b Dakora et al. (1987); Ojiem et al. (2007); Pule-Meulenberg and Dakora (2009); Nyemba and Dakora (2010); Mokgehle et al. (2014); Bado et al. (2006a); Jones (1974); MacColl (1989); Kaleem (1989); Ncube et al. (2007); Kaleem (1989).

c Oikeh et al. (2010); Osunde et al. (2003); Singh et al. (2003); Okogun et al. (2005); Ojiem et al. (2007); Laberge et al. (2009); Mapope and Dakora (2016); Sanginga (2003); MacColl (1989); Okereke and Eaglesham (1993); Kasasa et al. (1999); Sanginga et al. (2002); Zingore et al. (2008); Yusuf et al. (2009b); Thuita et al. (2012); Adeboye et al. (2006); Carsky et al. (1997); Carsky et al. (1999); Kaleem (1989); Mapope and Dakora (2016).

d Ncube et al. (2007); Chikowo et al. (2004); MacColl (1989); Cobbina (1995); Mapfumo et al. (1999); Marandu et al. (2013).

e Ojiem et al. (2007); Nyemba and Dakora (2010).

f Ojiem et al. (2007); MacColl (1989); Okogun et al. (2005).

g Pule-Meulenberg and Dakora (2009); Nyemba and Dakora (2010); Ncube et al. (2007); Kaleem (1989).

The belowground N contributions of legumes are often neglected in the calculation of N field balances of legume crops. The contribution of N through roots and rhizodeposits can be substantial, though the precise quantification is difficult (Wichern et al., 2008). In a review of literature, Wichern et al. (2008) found that on average 33% of the total plant N in legumes (average across different legume species) is found belowground with a range of 14 to 74%. A large part of that N, on average 64%, is found in rhizodeposits (exudates, sloughed off cells and fine roots). Nitrogen in rhizodeposits is readily available for plant uptake, while larger roots usually have high C-to-N ratios, decompose slowly and immobilise N over a prolonged period (e.g. Urquiaga et al. (1998)). Given that belowground plant-derived N of legumes usually remains in the field after crop harvest (with the exception of groundnut roots that may be removed from the field together with the stover), neglecting the belowground N contribution can substantially underestimate the field N balance of legumes. This could explain why the residual N effects of legumes can be larger than expected given the aboveground net N balance of the legumes. Belowground plant-derived N of soybean growing in the derived and Guinea savannah of West Africa was found to constitute 16% and 23% respectively of the total plant N, with 70% of the belowground N found in the rhizodeposits (Laberge et al., 2009). Adding belowground N to an N field balance of soybean, taking the N fixation rate into account and assuming stover remains in the field, changed the field balance from +2 to +13 kg N ha^−1^ in the derived savannah and from +56 to +95 kg N ha^−1^ in the northern Guinea savannah. Another study with cowpea in the Sudan savannah (Laberge et al., 2011) reported that 52% of cowpea-derived N was located belowground (which seems a rather high proportion), with 85% of this N in rhizodeposits. Adding belowground N to the N field balance changed the balance from +9 to +22 kg N ha^−1^. We found no other studies from SSA that quantified belowground N contributions of legumes.

#### 3.2.2 Phosphorus

An adequate supply of phosphorus is key for good growth and effective N_2_-fixation by legumes. While P fertiliser is needed in the P deficient soils of SSA, some legumes are able to mobilise less-labile forms of P compared with cereals. Recycling legume residues that contain P taken up by the legume can improve the P nutrition of a cereal in a cereal-legume rotation. Given that legume grain typically has a P concentration two to three times higher than that of the residues (Nijhof, 1987) and a harvest index of around 0.35 under normal growing conditions, about 35–50% of the P in the aboveground parts is present in crop residues. Whereas most research focused on leguminous cover crops, among the grain legumes, cowpea (Jemo et al., 2006, Pypers et al., 2006), soybean (Jemo et al., 2006), pigeonpea (Noriharu et al., 1990, Kamh et al., 2002, Richardson et al., 2009), chickpea (Richardson et al., 2009, Mat Hassan et al., 2012), faba bean (Mat Hassan et al., 2012) and white lupin (Gardner et al., 1983, Braum and Helmke, 1995, Mat Hassan et al., 2012) have been found to access less-labile forms of P under P-deficient conditions. Grain legumes acquire poorly available P through three main mechanisms: i) mobilisation of non-labile P through the release of root exudates; ii) access to more of the labile P through a finer root architecture, and iii) enhanced associations with mycorrhiza. Lupin is unique among grain legumes in having cluster roots (Neumann et al., 2000) and is not discussed further. In addition to accessing less-labile forms of P, legumes can improve P acquisition by maize through modification of soil biological and chemical properties.

Under P-deficient conditions, the roots of crops can release organic anions and organic acids that modify the chemistry of the rhizosphere and mobilise various forms of inorganic P. Exuded organic anions replace phosphate ions on sorption sites and mobilise P bound in humic-metal complexes (Richardson et al., 2009). In addition, Noriharu et al. (1990) and Kamh et al. (2002) showed that pigeonpea can take up P from Fe/Al-phosphates through exudation of piscidic and malonic acid. The exudation of organic acids also lowers the pH of the rhizosphere, which may increase the solubility of P in some soils. H^+^ released in the rhizosphere to counterbalance anions that leave decreases the pH of the soil solution (Hinsinger, 2001, Adeboye et al., 2006, Pypers et al., 2007, Richardson et al., 2009). Especially N_2_-fixing legumes were found to have a net positive excess of cations over anions entering their roots and are therefore expected to release more H^+^ than non-N_2_-fixing crops (Hinsinger, 2001). Faba bean, pigeonpea, chickpea, cowpea and white lupin have been found to release more and more effective organic acids than cereals (e.g.Gardner et al., 1983, Jemo et al., 2006, Pypers et al., 2006, Richardson et al., 2009). Another way in which plants can mobilise P is by increased release of extracellular phosphatases that mineralise soil organic P in the rhizosphere (Richardson et al., 2009). Kamh et al. (1999) and Kamh et al. (2002) related efficient P acquisition of pigeonpea to its large phosphatase activity. Jemo et al. (2006) related P-efficiency of soybean varieties to high activity of root surface phosphatases. The P mobilizing exudates released by legume roots turn over rapidly (Nuruzzaman et al., 2005). They can affect the P acquisition of an intercropped cereal, but it is unlikely that a subsequent cereal in a rotation directly benefits from P mobilizing root exudates. A change in pH, however, can affect the dissolution of P in a subsequent season, as hypothesised by Pypers et al. (2007).

With mechanisms similar to those used to access poorly available soil P, legumes can improve the utilisation of phosphate rock (PR) in a legume-cereal rotation under P-deficient conditions, leading to increased yield and P-uptake of a subsequent maize crop. *Mucuna pruriens* and lablab enhanced solubilisation of PR compared with maize (Vanlauwe et al., 2000, Pypers et al., 2007), probably due to decreases in soil pH induced by the legume (Pypers et al., 2007). Both studies report increases in the labile P pool of the topsoil after the legumes were grown, but the measures of P availability did not correspond with observed increases in yield and P uptake from the subsequent maize crop. Whereas Vanlauwe et al. (2000) concluded that legumes not only increase the immediately available P pool, but also less labile P pools, Pypers et al. (2007) suggest that the legume in the rotation has other positive, possible microbiological effects in the soil which enhanced maize growth.

Differences in root morphology may account for changes in P uptake. Nuruzzaman et al. (2005) found that although faba bean was better at taking up P from the soil than maize, faba bean did not exudate much organic anions. Instead, the extensive root system of faba bean explored a large volume of soil and was able to access more P (and other nutrients) than maize. Rose et al. (2010) found that the strong P uptake from P efficient faba bean genotypes in an acid soil was strongly correlated with root growth traits. In soybean, variability in P uptake efficiencies between varieties has been associated with differences in root hair development (length and density) (Vandamme et al., 2013).

Through associations with arbuscular mycorrhizal (AM) fungi, legume may access poorly available P. Differences in P uptake efficiency in soybean has been associated, to some extent, with colonisation by AM fungi (Vandamme et al., 2013). The cultivation of legumes in rotation with cereals may also lead to higher AM infection rates of cereal roots (see Section 3.3.3) which may help to enhance P uptake. Alvey et al. (2001) reported increased cereal shoot P concentrations and P uptake, together with higher AM infection rates in cereals following legumes, relative to continuous cereals in SSA.

The effectiveness of mechanisms in enabling efficient uptake of P depends on soil characteristics. Different varieties of the same species may exploit different mechanisms to take up P. In an acid soil, efficient P uptake from P efficient faba bean genotypes was strongly correlated with root growth traits. In an alkaline soil, P efficient varieties had the highest malate exudation (Muhr et al., 2002, Rose et al., 2010). Only those P-efficient varieties increased subsequent maize yield (Rose et al., 2010). Also Jemo et al. (2006) and Vandamme et al. (2013) reported that certain cowpea and soybean varieties are more efficient at taking up P than others.

#### 3.2.3 Other nutrients and pH

While the legume tree *Senna siamea* has shown the ability to recycle Ca from a subsoil to the top soil in the West African derived savannah (Vanlauwe et al., 2005), it is unlikely that shallowly rooting annual grain legumes have such abilities. Legumes take up nutrients other than N and P and thereby influence the availability of these nutrients to a subsequent cereal crop, depending on nutrient removal rates and crop residue management. While in theory it is possible that legumes affect the availability of nutrients other than N and P through changes in soil structure or soil microbial activities, we found no evidence for such mechanisms.

Legume have the ability to induce soil acidification (Haynes, 1983) (see also Section 3.2.2) and long-term legume pastures have been associated with declines in pH and increased levels of extractable manganese and aluminium potentially toxic to many agricultural plants (Bromfield et al., 1983). Soils that are already mildly acid and light-textured sandy soils are the most prone to acidification. While annual grain legumes also have a soil acidifying effect (Yan et al., 1996), a decline in soil pH should be less of a concern in an annual legume crop rotated with a cereal. Moreover, decomposition of legume residues can counteract soil acidification and even help to increase pH (Tang and Yu, 1999). As a result, legume-cereal rotations in SSA have been associated with a higher soil pH than continuous cereal rotation (Alvey et al., 2001, Marschner et al., 2004) with reported increases in pH between 0.1 and 1.3, though results are inconsistent with some reporting no changes in pH (Bationo and Ntare, 2000).

#### 3.2.4 Soil organic matter, soil structure and soil moisture

Compared with cereals, legume residues are relatively rich in N with a narrow C-to-N ratio. These characteristics favour rapid decomposition and release of N to subsequent crops. Further, the amount of residual biomass produced by grain legumes is usually less than that of cereals, giving less C input from grain legumes into the soil. Comparisons of soil C contents after legume-cereal rotations with continuous cereal cropping in SSA generally do not show significant differences (Bationo and Ntare, 2000, Franke et al., 2008, Franke et al., 2010, Yusuf et al., 2009a, Anyanzwa et al., 2010), although this may be due to the limited duration (<4 years) of most trials. The main plausible way through which the incorporation of grain legumes into cereal-based system could enhance soil C contents is through an enhanced productivity of a subsequent cereal crop, or through intercropping systems with enhanced total biomass production (Samaké et al., 2006). Indirectly, the availability of legume residues at smallholder farms can facilitate integrated crop-livestock production, offering incentives for improved conservation of residues over dry seasons and enhanced C inputs through animal manure, especially in arid areas where most organic matter on the soil surface after crop harvest disappears during the dry season (Franke et al., 2008). Enhancement of soil organic matter contents after grain legumes may results in changes in soil structure and soil moisture availability and in turn on yields of subsequent crops. Evidence for direct effects of legumes on soil structure is scarce. Mytton et al. (1993) observed an improvement in crumb structure of clay soils under grass-clover mixtures compared with pure grass swards in Wales. Grain legumes have been associated with a reduced water uptake and sparing of soil water, leading to increased water availability in a subsequent cereal, relative to continuous cereals (Miller et al., 2002). We found no studies observing this in SSA, and this effect may not be relevant in large parts of SSA that have long dry seasons during which most soil available water gets depleted in the rooted zone. Ncube et al. (2007) reported that grain legumes in a field experiment in Zimbabwe used more soil water than sorghum at a depth of 0–55 cm, but differences in soil moisture disappeared after the first single large rain event at the start of the next growing season.

#### 3.2.5 Allelopathy

The only case we could identify where deleterious residual impacts of legumes have been attributed to allelopathy is with mung bean (*Vigna radiata)* in continuous cropping (Waller et al., 1994) or on other crops (Lertmongko et al., 2011). There appears to be no evidence of allelopathic effects with other grain legumes.

### 3.3 Biotic factors

#### 3.3.1 Weeds

Green manure legumes, such as *Mucuna pruriens, Pueraria phaseoloides* and *Desmodium* spp., and fodder legumes, such as *Stylosanthes guianensis*, have the ability to effectively suppress obnoxious weeds in the derived savannah of West Africa (Chikoye and Ekeleme, 2003) and in East Africa (Kifuko-Koech et al., 2012). Grain legumes tend not to have the same weed-suppressing ability (Chikoye et al., 2008). In high input systems relying on herbicides for weed control, incorporating grain legumes into maize-based systems allows farmers to use herbicides with different modes of action, although this benefit is lost if glyphosate-resistant crops are grown continuously. As smallholders typically rely on weeding by hand or through animal traction, legume crops with enhanced weed-suppressing ability relative to other crops would be advantageous, but there is little evidence for this. However, legumes can be of great benefit in reducing the impact of parasitic weeds. In SSA, the obligate parasitic weeds *Striga asiatica* or *S. hermonthica* (both referred to as striga below) are widespread and cause massive yield loss in cereals.^1^

Legumes are non-hosts of striga and appear to trigger ‘suicidal’ germination of striga seed, thereby reducing the seedbank directly and preventing replenishment of the seedbank, while the longevity of striga seed in moist soil appears to be limited (Gbèhounou et al., 2003). Signalling molecules excreted by plant roots called strigolactones induce the germination of obligate parasitic plants such as striga, as well as the growth of beneficial AM fungi, that help the plant to access poorly-available soil P (Bouwmeester et al., 2007). A wide range of plant species including legumes excretes strigolactones. The presence of strigolactones can lead to suicidal germination of striga seed when excreted by a non-host, as striga needs to germinate within the close vicinity of the roots of a host plant to survive. The excretion of strigolactones is stimulated by P deficiencies. Plant exudates usually contain more than one strigolactone and differences in composition exist even within varieties (Awad et al., 2006), creating possibilities for breeders to screen legumes varieties for their ability to stimulate suicidal germination. However, given the multiple roles of strigolactones as signalling molecules and as growth regulators within the plant and the influence of the environment on the excretion of strigolactones (Ruyter-Spira et al., 2013), screening and selecting striga varieties for this ability is likely to be difficult. The reduction in striga seedbank resulting from a legume crop can strongly benefit the yield of a subsequent cereal crop (Ellis-Jones et al., 2004, Franke et al., 2006, Rusinamhodzi et al., 2012). Natural fallows often harbour grass weeds that can serve as a host of striga attacking cereals, allowing striga to set seed and replenish its seed bank. Legume-cereal intercropping can help in suppressing striga, but the continued cultivation of a cereal in the field provides a host for striga (Carsky et al., 1994, Oswald, 2005, Kifuko-Koech et al., 2012, Midega et al., 2014). The boost in soil fertility from growing legumes also plays a strong role in rendering cereal crops less susceptible to damage by striga.

#### 3.3.2 Pests and diseases

A reduced pest and diseases pressure for cereal crops is frequently mentioned as a key reason to rotate with grain legumes. Evidence of such benefits in SSA however is surprisingly limited. Rotating maize with cowpea or soybean has been found to increase attacks by stem borer (*Busseola fascu*) in maize in southern Cameroon (Chabi-Olaye et al., 2005a). The improved nutritional status of maize plants rotated with legumes appeared to increase attacks, but also improved plant vigour resulting in a net benefit for the plant. Intercropping cereals with non-host legumes, such as lablab, cowpea, soybean, or other non-host crops, such as cassava, does help to reduce attacks by Lepidopterous stem borer including *Busseola fascu* in different parts of SSA (Skovgård and Päts, 1996, Chabi-Olaye et al., 2005b, Maluleke et al., 2005), most likely by disrupting host-plant finding (Calatayud et al., 2014).

The best studied impacts of legumes on pests and diseases concern impacts on nematodes. Nematodes can substantially limit cereal productivity and nematode population density and damage potential increase with cereal cropping intensity (Coyne and Plowright, 2000, Talwana et al., 2008). Although a wide range of nematode pests is associated with cereals, only a few are of economic importance, depending on geography and circumstances. In SSA, various studies confirm the negative impact of nematodes on maize, mostly *Pratylenchus* spp. and *Meloidogyne* spp. (Afolami and Fawole, 1991, Kagoda et al., 2011). These nematodes however tend to have wide host ranges including legumes and various weed species, limiting the possibilities to use rotational legumes to control them (Viaene et al., 2013). Moreover, these nematodes can damage legumes’ growth abilities as well. *Meloidogyne* spp. for instance has been found to reduce soybean’s ability to fix atmospheric N_2_ (Coyne and Oyekanmi, 2007).

Studies from SSA (all conducted in West Africa) do not provide a consistent picture regarding grain legumes’ ability to suppress parasitic nematodes. Bagayoko et al. (2000) observed that groundnut reduces parasitic nematode populations in a groundnut/sorghum intercrop systems, relative to sole sorghum in the Sudano-Sahelian zone, whereas sole groundnut had the lowest nematode population densities, indicating groundnut is a poor host for the nematode groups assessed (*Helicotylenchus* sp., *Rotylenchus* sp. and *Pratylenchus* sp.). Cowpea intercropped with millet on the other hand did not appear to suppress nematodes or even exacerbated nematode pressure in millet. This is in line with Bado et al. (2011) who found that cowpea-sorghum rotations increased nematode counts in sorghum by 1.5–2.0 times, while groundnut-sorghum rotation reduced counts in the sorghum rhizosphere and roots 17 to 19 times, relative to continuous sorghum. Alvey et al. (2001) reported 60–80 times more nematodes in roots of continuously-cropped millet than that in roots of millet following cowpea or groundnut in the Sudano-Sahelian zone. They did not distinguish between the two legume crops. Marschner et al. (2004) on the other hand found inconsistent effects of groundnut on nematode densities in rotational maize and sorghum crops, relative to continuous cereal, in soils from the Sudan and the Guinea savannah.

#### 3.3.3 Other effects on the soil microbiota

DNA techniques such as PCR-DGGE have shown that bacterial species composition in soil under rotation is clearly distinct from soils under continuous cereal cultivation in SSA (Alvey et al., 2003, Marschner et al., 2004). Grain legume-cereal systems in SSA have been associated with changes such as higher AM infection rates (Bagayoko et al., 2000, Alvey et al., 2001) with up to 20% more roots infected, higher soil bacterial C and N levels (Marschner et al., 2004, Adeboye et al., 2006, Yusuf et al., 2009a), a lower microbial biomass C-to-N ratio (Marschner et al., 2004) and a higher fungal biomass (Adeboye et al., 2006) in the rhizosphere of cereals, relative to continuous cereals. Apart from direct effects on symbionts such as rhizobia or mycorrhiza that for instance help plants to access soil P (see Section 3.2.2), it is unclear what changes in soil microbe populations contribute to cereal growth (Marschner et al., 2004).

## 4 Final remarks and recommendations

Our review of the literature shows that rotational benefits of grain legumes on cereal yields are substantial and widespread. While there may be a positive bias in reporting, the consistent responses at multiple sites differing in climate or soil fertility provide strong support that cereal yields are enhanced after grain legumes. The residual effects of grain legumes should take a prominent place when evaluating the pros and cons of sustainable intensification with grain legumes. We did not consider some of the wider cropping and farming system effects of grain legumes in this review. For instance, grain legumes can make up an important component of an ISFM strategy increasing overall crop productivity and soil fertility beyond N benefits (Vanlauwe et al., 2010, Vanlauwe et al., 2015). While grain legumes on their own are unlikely to increase soil organic matter contents (see Section 3.2.4), increased residue inputs as a result of a successful ISFM strategy can do so. Residues of grain legumes also play an important role in integrated crop-livestock systems, which offer opportunities and challenges to optimise natural resource use and organic input applications to crops (Duncan et al., 2016). We did not consider grain legume-cereal intercropping systems which are common in parts of SSA (e.g. common bean-maize mixed cropping in East Africa and cowpea relay cropping in cereals in West Africa). While the N_2_-fixing capacities of grain legumes are maintained in an intercrop, though at a lower rate due to competition with the cereal, the benefits of grain legumes as a non-host of pests, diseases and striga in cereals could be strongly reduced.

Future research on N_2_-fixation by grain legumes and residual N benefits in SSA should focus on explaining the wide variability observed between sites. We retrieved only two studies that assessed residual benefits on a relatively large (>15) number of farmers’ fields (Kolawole et al., 2007, van Vugt et al., 2017) and only the latter study attempted to disentangle the factors behind the observed variability. Measuring the rotational effects along with the relevant covariates on a large number of farmers’ fields, for instance through simple two-treatment (legume-cereal vs. continuous cereal) comparisons (Vanlauwe et al., in press), would provide better understanding of the sources of variability in rotational effects under farmers’ diverse environmental and socio-economic conditions (e.g. see Ronner et al. (2016) on legume responses to inputs). As there is a fair understanding of mechanisms how grain legumes can affect P availability in the subsequent cereal, future research should focus on assessing if and where these mechanisms are relevant in farmers’ fields. The other non-N benefits of grain legumes are still poorly quantified and understood in SSA, with the exception of legumes’ impact on striga population dynamics. Clearly, there is a need for more detailed mechanistic studies to highlight non-N effects, particularly in relation to common pests and diseases in cereals. Also in relation to biotic factors, a key question is if and under which conditions benefits of enhanced pest and disease suppression can be expected to occur in farmers’ fields.

## Footnotes

1 *Striga gesnerioides* attacks cowpea and is not discussed here.
